# Absorption in narrow and wide gap materials

**DOI:** 10.1016/j.heliyon.2023.e21507

**Published:** 2023-10-27

**Authors:** Aneer Lamichhane

**Affiliations:** Western Colorado University, Department of Natural and Environmental Sciences, Hurst Hall 110, Gunnison, 81231, CO, United States of America

**Keywords:** Absorption line width, Penn model, Dielectric function

## Abstract

The absorption spectrum of a material reveals the absorbed light frequencies, characteristic peaks, and the line width of absorption bands. This information is critical for understanding the energy levels involved in the absorption process as well as the material's electronic structure. In this study, an equation connecting the absorption line width with the static dielectric function is derived for narrow and wide gap materials. It is then compared with the Penn model. It has been found that the constant in the Penn model has a value that is restricted to the range of 0.5 to 1. Application of this equation to various narrow and wide gap materials is then discussed.

## 1 Introduction

In spectroscopy, the line width is related to the form and width of the absorption line. Materials with a wider line width can absorb light at a wide range of frequencies, while materials with a narrower line width absorb light at a specific frequency. In the case of narrow and wide gap materials, the absorption spectra of the oscillator can be approximated by the following isotropic energy-band model [1],

(1) $$\epsilon_{2}(\omega )=\left\{ \begin{array}{cc} 0, & 0\leq \hbar \omega \leq E_{g}\\ \frac{16}{3a_{o}k_{F}}\frac{E_{F}^{2}E_{g}^{2}}{{\left(\hbar \omega \right)}^{4}}\frac{\hbar \omega }{\sqrt{\left[{\left(\hbar \omega \right)}^{2}-E_{g}^{2}\right]}}, & E_{g}\leq \hbar \omega \end{array}\right.$$

where $a_{o}=\frac{\hbar^{2}}{me^{2}}$ is the Bohr radius, $k_{F}$ and $E_{F}$ are the Fermi wave number and Fermi energy, respectively. Using the Kramers-Kronig relation, Equation (1) can be transformed at ω=0, as illustrated by Phillips [2],

(2) $$\epsilon_{1}(0)=1+{\left(\frac{\hbar \omega_{p}}{E_{g}}\right)}^{2}S_{o}$$

where $\omega_{p}$ is the plasma frequency and Equation (2) is the well-known Penn model, and the average gap $E_{g}$ is sometimes, known as the Penn gap, and $S_{o}$ as the Penn constant. Penn [3], in 1962, derived the wave vector dependent dielectric function in semiconductors by undertaking the formation of standing waves in the Brillouin zone and the Umklapp process, and the consequence of his result at zero wave vector is,

(3) $$\epsilon_{1}(0)=1+{\left(\frac{\hbar \omega_{p}}{E_{g}}\right)}^{2}\left[1-\frac{E_{g}}{4E_{F}}+\frac{1}{3}{\left(\frac{E_{g}}{4E_{F}}\right)}^{2}\right]$$

Penn further considered $\frac{E_{g}}{E_{F}}$ as small neglected quantity, and approximated the bracketed term in Equation (3) to be nearly unity, and therefore, $S_{o}=1$ in the Penn original formalism. Grimes and Cowley [4], in 1975, pointed out various approximations used in the Penn model such as isotropic nearly free electron model, replacing the squared matrix element by its value on the Fermi surface, dielectric function not defined for certain wave vector ranges; all of these could have contributed to discrepancy in the Penn formalism. To avoid unnecessary approximations, Grimes and Cowley numerically evaluated the dielectric function, and shown $S_{o}=0.62$ can be considered the representative value, and their results are in fairly good accord with pseudopotential calculations. Later Cirilo-Lombardo [5], in 1980, improved the Penn model by taking excitons into account, and shown analytically that the dielectric function is $\frac{2}{3}$ times as the Penn model predicted,

(4) $$\epsilon_{1}(0)=1+\frac{2}{3}{\left(\frac{\hbar \omega_{p}}{E_{g}-E_{x}}\right)}^{2}\left[1-\frac{E_{g}-E_{x}}{4E_{F}}+\frac{1}{3}{\left(\frac{E_{g}-E_{x}}{4E_{F}}\right)}^{2}\right]$$

where $E_{x}$ is the Wannier exciton binding energy, and under $E_{g}>E_{x}$, the analytically found $S_{o}=\frac{2}{3}$ is close to the numerical value found by Grimes and Cowley.

Modeling the absorption spectrum by the Lorentz single oscillator model,

(5) $$\epsilon_{2}(\omega )=\frac{\omega_{p}^{2}\gamma \omega }{{\left(\omega_{o}^{2}-\omega^{2}\right)}^{2}+\gamma^{2}\omega^{2}}$$

where *γ* is the line width proportional to the damping constant, can directly lead to,

(6) $$\epsilon_{1}(0)=1+{\left(\frac{\hbar \omega_{p}}{E_{o}}\right)}^{2}$$

If $E_{o}=\hbar \omega_{o}$ is considered as the dominant resonance frequency then one can approximate it with the Penn gap, i.e., $E_{o}\approx E_{g}$. This is because the Penn gap in most narrow and wide gap materials is found to lie in the UV regions [6]. This naturally suggests that modeling the absorption spectra of materials using Lorentz single dominant oscillator yields a decent result. This has already been shown true by the Wemple - DiDomenico [7], [8] formalism in several narrow and wide gap materials. However there are still some unexplored questions, such as: Can one define $S_{o}$ from the Lorentz single dominant oscillator model? If so, what may the value of $S_{o}$ be? It would either be $S_{o}=1$, as was suggested by Penn's, or something else. Is there a relationship between the width of the absorption line produced by the Lorentz dominant oscillator with $S_{o}$? This study attempts to provide these answers.

## 2 Theory

The optical absorption of solids can be described by the complex dielectric function $\epsilon_{1}+i\epsilon_{2}$, where $\epsilon_{1}$ and $\epsilon_{2}$ are the real function of the frequency *ω*. The functions $\epsilon_{1}(\omega )$ and $\epsilon_{2}(\omega )$ are connected by the Kramers-Kronig relation,

(7) $$\epsilon_{1}(\omega )=1+\frac{2}{\pi }\int_{0}^{\infty }\frac{\epsilon_{2}(\omega^{\prime })}{\omega^{\prime }-\omega }d\omega^{\prime }$$

One good approximation to consider is that $\epsilon_{2}(\omega^{\prime })=0$ outside of this interval $\left[\omega_{0}-\frac{\gamma }{2},\omega_{0}+\frac{\gamma }{2}\right]$. That means replacing the full absorption structure by a single oscillator centered at the resonance frequency $\omega_{0}$. With this approximation and concerning the static dielectric constant for non-conductive solids,

(8) $$\epsilon_{1}(0)=1+\frac{2}{\pi }\int_{\omega_{0}-\frac{\gamma }{2}}^{\omega_{0}+\frac{\gamma }{2}}\frac{\epsilon_{2}(\omega^{\prime })}{\omega^{\prime }}d\omega^{\prime }$$

By using the Riemann sum, one can approximate the definite integral,

(9) $$\int_{\omega_{0}-\frac{\gamma }{2}}^{\omega_{0}+\frac{\gamma }{2}}\frac{\epsilon_{2}(\omega^{\prime })}{\omega^{\prime }}d\omega^{\prime }=\lim_{n\to \infty }\sum_{k=1}^{n}\frac{\epsilon_{2}(c_{k})}{c_{k}}\Delta \omega^{\prime }$$

where $c_{k}=\omega_{0}-\frac{\gamma }{2}+(k-1)\Delta \omega^{\prime }$, and $\Delta \omega^{\prime }=\frac{\gamma }{n}$. Furthermore defining $\epsilon_{2}$ by the Lorentz single oscillator model,

(10) $$\epsilon_{2}(c_{k})=\frac{\omega_{p}^{2}\gamma c_{k}}{{\left(\omega_{0}^{2}-c_{k}^{2}\right)}^{2}+\gamma^{2}c_{k}^{2}}$$

results in,

(11) $$\lim_{n\to \infty }\sum_{k=1}^{n}\frac{\epsilon_{2}(c_{k})}{c_{k}}\Delta \omega^{\prime }=\lim_{n\to \infty }\frac{\omega_{p}^{2}\gamma^{2}}{n}\sum_{k=1}^{n}\frac{1}{{\left(\omega_{0}^{2}-c_{k}^{2}\right)}^{2}+\gamma^{2}c_{k}^{2}}$$

As it has shown in Appendix A and Appendix B, a complete mathematical formulation, that enables us to write,

(12) $$\lim_{n\to \infty }\sum_{k=1}^{n}\frac{1}{{\left(\omega_{0}^{2}-c_{k}^{2}\right)}^{2}+\gamma^{2}c_{k}^{2}}=\frac{\pi }{2}\frac{n}{{\left(\omega_{0}^{2}-c_{1}^{2}\right)}^{2}+\gamma^{2}c_{1}^{2}}$$

Under the approximation, $c_{1}=\omega_{0}-\frac{\gamma }{2}\approx \omega_{0}$ yields,

(13) $$\lim_{n\to \infty }\sum_{k=1}^{n}\frac{\epsilon_{2}(c_{k})}{c_{k}}\Delta \omega^{\prime }=\frac{\pi }{2}\frac{\omega_{p}^{2}}{\omega_{0}^{2}}$$

Equations (8), (9) and (13) results,

(14) $$\epsilon_{1}(0)=1+\frac{\omega_{p}^{2}}{\omega_{0}^{2}}$$

which is the same equation, as mentioned in Equation (6). In most solids due to interaction between atoms, and the nature of the energy bands of electrons, the damping forces are present. Under such condition, simplifying Equation (12) by substituting $c_{1}=\omega_{0}-\frac{\gamma }{2}$, without approximation, yields,

(15) $$\lim_{n\to \infty }\sum_{k=1}^{n}\frac{\epsilon_{2}(c_{k})}{c_{k}}\Delta \omega^{\prime }=\frac{\pi }{2}\frac{\omega_{p}^{2}}{\omega_{0}^{2}}{\left[2-\frac{3}{2}\frac{\gamma }{\omega_{0}}+\frac{5}{16}\frac{\gamma^{2}}{\omega_{0}^{2}}\right]}^{-1}$$

Equations (8), (9) and (15) results,

(16) $$\epsilon_{1}(0)=1+\frac{\omega_{p}^{2}}{\omega_{0}^{2}}{\left[2-\frac{3}{2}\frac{\gamma }{\omega_{0}}+\frac{5}{16}\frac{\gamma^{2}}{\omega_{0}^{2}}\right]}^{-1}$$

Comparing it with the Penn model,

(17) $${\left[2-\frac{3}{2}\frac{\gamma }{\omega_{0}}+\frac{5}{16}\frac{\gamma^{2}}{\omega_{0}^{2}}\right]}^{-1}=1-\frac{E_{p}}{4E_{F}}+\frac{1}{3}{\left(\frac{E_{p}}{4E_{F}}\right)}^{2}$$

Equation (17) produces a quadratic equation, and under the condition $\frac{\gamma }{\omega_{0}}<1$, results in the final expression,

(18) $$\frac{\gamma }{\omega_{0}}=\frac{4}{5}\left(3-\sqrt{\frac{5}{S_{o}}-1}\right)$$

Equation (18) shows the necessary connection between the line width of the Lorentz single dominant oscillator with $S_{o}$.

## 3 Results and discussions

**Figure 1:**
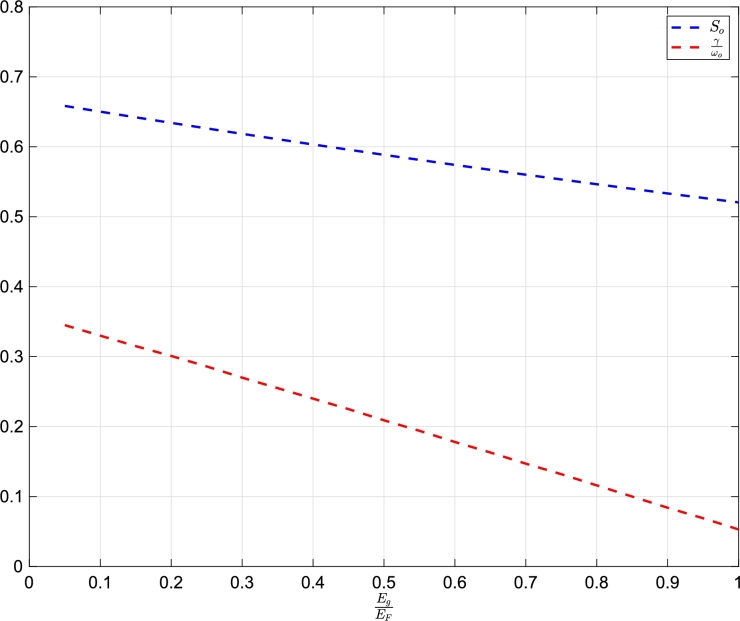
Variation of *S*_*o*_ and $\frac{\gamma }{\omega_{o}}$ as a function of $\frac{E_{g}}{E_{F}}$. Appendix C contains their data.

Equation (18) shows that $S_{o}$ is related to the absorption spectra of materials. As $S_{o}$ lowers, the line width per resonance reduces as well. For the same maximum kinetic energy of valence electrons, a higher value of $S_{o}$ implies low band gap materials, and the band gap widens as $S_{o}$ decreases. As a result, the broader line widths in the absorption spectra tend to diminish, as shown in Fig. 1. In the next, $S_{o}$ of different narrow and wide gap materials is calculated. At first, $\frac{\gamma }{\omega_{o}}$ is calculated as,

(19) $$\frac{\gamma }{\omega_{o}}=\frac{1}{A}\frac{\omega_{p}^{2}}{\omega_{o}^{2}}$$

where *A* is the amplitude of oscillation. Then using Equation (18), the required $S_{o}$ is calculated as shown in Table 1.

**Table 1:** Calculated $\frac{\gamma }{\omega_{o}}$ and *S*_*o*_ values for various narrow and wide gap materials.

	*E*_*g*_ (eV)^1^	*ω*_*p*_ (eV)^1^	*A*	$\frac{\gamma }{\omega_{o}}$	*S*_*o*_
C	13.5	31.20	18.34^2^	0.29	0.63
Si	4.77	16.60	45.35^3^	0.27	0.62
Ge	4.31	15.60	30.09^3^	0.43	0.71
GaP	5.75	17.10	26.58^3^	0.33	0.65
GaAs	5.20	15.60	25.19^3^	0.36	0.66
GaSb	4.12	16.70	25.14^3^	0.65	0.86
InP	5.16	17.80	22.95^3^	0.52	0.76
InAs	4.58	13.85	22.17^3^	0.41	0.69
InSb	3.73	12.70	21.17^3^	0.55	0.78
AlSb	4.14	13.91	25.97^4^	0.43	0.71
PbSe	3.17	15.37	33.45^5^	0.70	0.90
ZnS	7.85	16.70	9.63^6^	0.47	0.73
ZnSe	7.05	15.60	11.74^7^	0.42	0.70
CdS	7.11	14.88	6.64^8^	0.66	0.87
CdSe	6.58	14.19	7.97^9^	0.58	0.81
CdTe	5.79	13.10	11.47^10^	0.45	0.72

1 Here's the second and third columns, from Ref. [9], [10].

2 Data, from [11].

3 Data, from [12].

4 Data, from [13].

5 Data, from [14].

6 Data, from [15].

7 Data, from [16].

8 Data, from [17].

9 Data, from [18].

10 Data, from [19].

$S_{o}=1$ may not be achieved for narrow and wide gap materials. Equation (14) demonstrates that the approximation $c_{1}=\omega_{0}-\frac{\gamma }{2}\approx \omega_{0}$ generates $S_{o}=1$. This approximation is true when electrons are either free to flow, so γ=0, or when $\omega_{0}>>\gamma$, indicating that the resonant frequency coincides with its natural frequency (plasma frequency). On the other hand, the approximation $c_{1}=\omega_{0}-\frac{\gamma }{2}\approx \omega_{0}$ is not appropriate in the case of narrow and wide gap materials since there are damping forces present that naturally reduce its natural frequency to resonance frequency. As a result, $S_{o}<1$ and its value are confined between 0.5 and 1. As long as the Fermi energy remains same, materials with $S_{o}$ near to 0.5 should be wide gap materials, while materials with $S_{o}$ close to unity should be narrow gap materials. In other words, $S_{o}$ near to 0.5 has a narrow line width corresponding to well-defined and isolated energy levels, whereas $S_{o}$ close to unity has a broader line width corresponding to maximal interaction or overlap between neighboring states. As a result, Equation (18) is necessary to comprehend the relationship between the Penn constant and the line width in order to comprehend absorption in narrow and wide gap materials.

## 4 Conclusions

For narrow and wide gap materials, this study constructs an equation relating the absorption line width with the static dielectric function. Because it is equivalent to the Penn model, it is compared to it, and the relationship of $S_{o}$ with $\frac{\gamma }{\omega_{o}}$ is demonstrated. The relationship is further examined among several narrow and wide band gap materials, and the results agree well, according to the band structure model. It is hoped that this study is fundamental to our understanding of the electronic and optical properties of materials.

## CRediT authorship contribution statement

**Aneer Lamichhane:** Writing – review & editing, Writing – original draft, Visualization, Formal analysis, Data curation, Conceptualization.

## Declaration of Competing Interest

The author declares that there is no known competing financial interests or personal relationships that could have appeared to influence the work reported in this paper.

## Figures and Tables

**Figure B.2:**
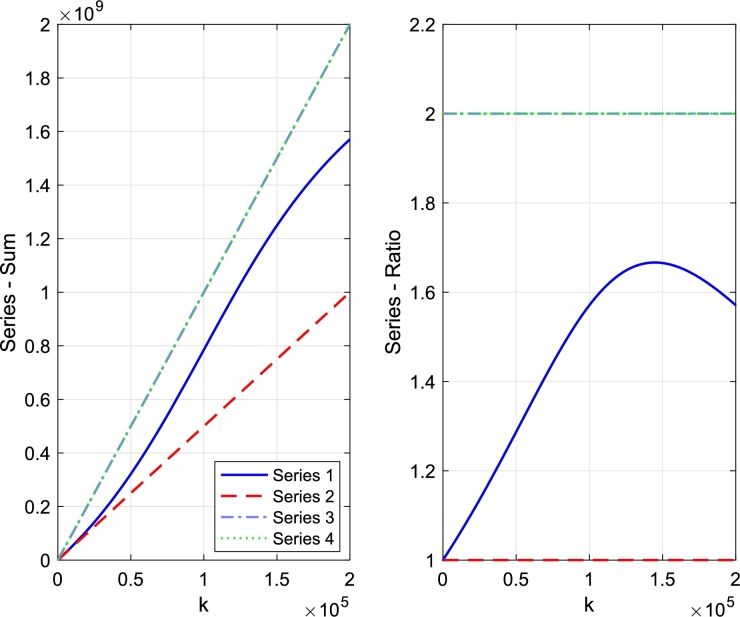
Calculated series-sum and series ratio as illustrated by inequalities (A.2), and (A.7). Series 3 and Series 4 are almost coincident suggesting *ϵ*_1_ and *ϵ*_2_ are infinitesimally small numbers.

**Figure B.3:**
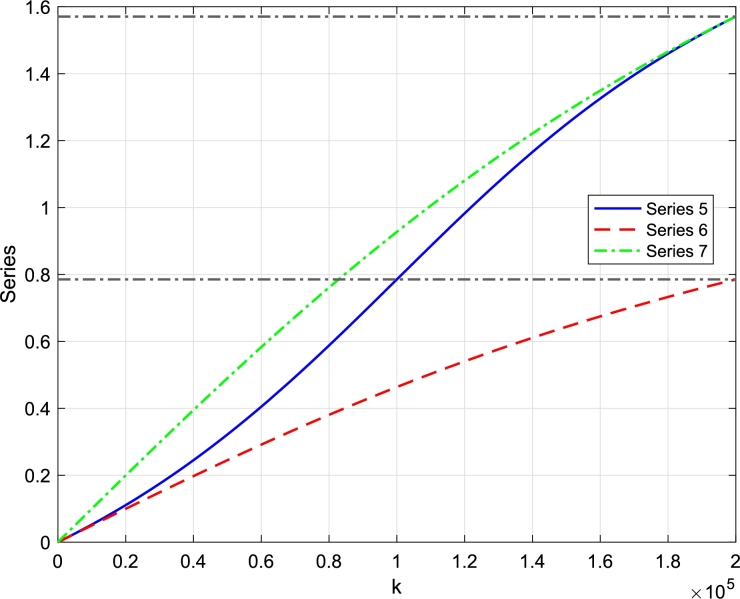
Calculated series-sum as illustrated by Series 5, 6, and 7. The upper and lower horizontal lines correspond to $\frac{pi}{2}$ and $\frac{pi}{4}$, respectively.

**Figure B.4:**
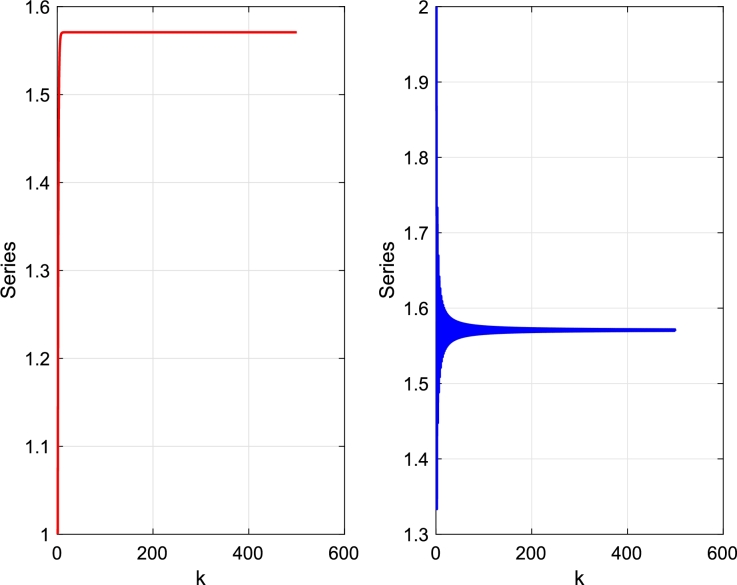
Series $\sum_{k=1}^{\infty }\{\sum_{r=0}^{k-1}\frac{2^{k-1}{\left(-1\right)}^{r}}{k+r}\left(\begin{array}{c} k-1\\ r \end{array}\right)\}$ shown with red color and Gregory - Leibniz - Madhava series $\sum_{k=1}^{\infty }2\frac{{\left(-1\right)}^{k+1}}{2k-1}$ shown with blue color. Both are computed for k=1 to 500.

**Table C.2:** Values of *S*_*o*_ computed by Grimes and Cowley [4] as a function of $\frac{E_{g}}{E_{F}}$. Values of $\frac{\gamma }{\omega_{o}}$ computed using Equation (18).

$\overset{¯}{E_{g}}$^1^	$S_{o}$	$\frac{\gamma }{\omega_{o}}$
0.05	0.6584	0.345
0.10	0.6502	0.330
0.15	0.6421	0.315
0.20	0.6342	0.301
0.25	0.6263	0.286
0.30	0.6185	0.270
0.35	0.6109	0.255
0.40	0.6033	0.240
0.45	0.5959	0.225
0.50	0.5885	0.209
0.55	0.5813	0.194
0.60	0.5741	0.178
0.65	0.5671	0.163
0.70	0.5601	0.147
0.75	0.5533	0.132
0.80	0.5465	0.116
0.85	0.5399	0.100
0.90	0.5333	0.084
0.95	0.5269	0.069
1.00	0.5205	0.053

1 is the reduced band gap, $\frac{E_{g}}{E_{F}}$.

## Data Availability

The sources of data have been mentioned in the references [4], [9], [11], [12], [13], [14], [15], [16], [17], [18], [19].

## Appendix A Analytical proof

Here, it will be shown analytically that,

(A.1) $$\frac{\lim_{n\to \infty }\sum_{k=1}^{n}\frac{1}{{\left(x^{2}-x_{k}^{2}\right)}^{2}+\alpha^{2}x_{k}^{2}}}{\lim_{n\to \infty }\sum_{k=1}^{n}\frac{1}{{\left(x^{2}-x_{0}^{2}\right)}^{2}+\alpha^{2}x_{0}^{2}}}=\frac{\pi }{2}$$

Let f be a real-valued function defined on the interval $\left[x-\frac{\alpha }{2},x+\frac{\alpha }{2}\right]$ where *x*, *α* ∈ $R_{\neq 0}$ such that x>>α. The interval $\left[x-\frac{\alpha }{2},x+\frac{\alpha }{2}\right]$ is partitioned into *n* sub-intervals: $x-\frac{\alpha }{2}=x_{0}<x_{1}<x_{2}<.....<x_{n-1}<x_{n}=x+\frac{\alpha }{2}$ of equal length $\frac{\alpha }{n}$. The sum of *f* is then defined as,

$$f_{n}=\sum_{k=1}^{n}\frac{1}{{\left(x^{2}-x_{k}^{2}\right)}^{2}+\alpha^{2}x_{k}^{2}},\forall x_{k}\in [x-\frac{\alpha }{2},x+\frac{\alpha }{2}].$$

Then it can be seen that,

(A.2) $$\sum_{k=1}^{n}\frac{1}{{\left(x^{2}-x_{0}^{2}\right)}^{2}+\alpha^{2}x_{0}^{2}}\leq \sum_{k=1}^{n}\frac{1}{{\left(x^{2}-x_{k}^{2}\right)}^{2}+\alpha^{2}x_{k}^{2}}\leq \sum_{k=1}^{n}\frac{1}{\alpha^{2}x^{2}}\leq \sum_{k=1}^{n}\frac{1}{\alpha^{2}x_{0}^{2}}\;or,1\leq \frac{\sum_{k=1}^{n}\frac{1}{{\left(x^{2}-x_{k}^{2}\right)}^{2}+\alpha^{2}x_{k}^{2}}}{\sum_{k=1}^{n}\frac{1}{{\left(x^{2}-x_{0}^{2}\right)}^{2}+\alpha^{2}x_{0}^{2}}}\leq \frac{\sum_{k=1}^{n}\frac{1}{\alpha^{2}x^{2}}}{\sum_{k=1}^{n}\frac{1}{{\left(x^{2}-x_{0}^{2}\right)}^{2}+\alpha^{2}x_{0}^{2}}}\leq \frac{\sum_{k=1}^{n}\frac{1}{\alpha^{2}x_{0}^{2}}}{\sum_{k=1}^{n}\frac{1}{{\left(x^{2}-x_{0}^{2}\right)}^{2}+\alpha^{2}x_{0}^{2}}}$$

Now let us evaluate the third term of the inequality,

(A.3) $$\frac{\sum_{k=1}^{n}\frac{1}{\alpha^{2}x^{2}}}{\sum_{k=1}^{n}\frac{1}{{\left(x^{2}-x_{0}^{2}\right)}^{2}+\alpha^{2}x_{0}^{2}}}=\frac{{\left(x^{2}-x_{0}^{2}\right)}^{2}+\alpha^{2}x_{0}^{2}}{\alpha^{2}x_{0}^{2}}$$

Using the relation $x=x_{0}+\frac{\alpha }{2}$; one can solve Equation (A.3) to get,

(A.4) $$\frac{\sum_{k=1}^{n}\frac{1}{\alpha^{2}x^{2}}}{\sum_{k=1}^{n}\frac{1}{{\left(x^{2}-x_{0}^{2}\right)}^{2}+\alpha^{2}x_{0}^{2}}}=2-\frac{3\alpha }{2x}+\frac{5\alpha^{2}}{16x^{2}}$$

Since x>>α and $\frac{3\alpha }{2x}>\frac{5\alpha^{2}}{16x^{2}}$, the right hand term can be expressed as $2-\epsilon_{1}$, where $\epsilon_{1}$ is the small number. Thus the third term becomes,

(A.5) $$\frac{\sum_{k=1}^{n}\frac{1}{\alpha^{2}x^{2}}}{\sum_{k=1}^{n}\frac{1}{{\left(x^{2}-x_{0}^{2}\right)}^{2}+\alpha^{2}x_{0}^{2}}}=2-\epsilon_{1}$$

Similarly, the last term of the inequality can be expressed as,

(A.6) $$\frac{\sum_{k=1}^{n}\frac{1}{\alpha^{2}x_{0}^{2}}}{\sum_{k=1}^{n}\frac{1}{{\left(x^{2}-x_{0}^{2}\right)}^{2}+\alpha^{2}x_{0}^{2}}}=2+\epsilon_{2}$$

where $\epsilon_{2}=\frac{\alpha }{2x_{0}}+\frac{\alpha^{2}}{16x_{0}^{2}}$; is another small number. Thus, in general, the inequality can be expressed as,

(A.7) $$1\leq \frac{\sum_{k=1}^{n}\frac{1}{{\left(x^{2}-x_{k}^{2}\right)}^{2}+\alpha^{2}x_{k}^{2}}}{\sum_{k=1}^{n}\frac{1}{{\left(x^{2}-x_{0}^{2}\right)}^{2}+\alpha^{2}x_{0}^{2}}}\leq 2$$

In the next, let us calculate the second term of this inequality beginning from the numerator of this term. Using $x_{k}=x_{0}+(k-1)\Delta x$, one can write,

(A.8) $$\sum_{k=1}^{n}\frac{1}{{\left(x^{2}-x_{k}^{2}\right)}^{2}+\alpha^{2}x_{k}^{2}}=\sum_{k=1}^{n}\frac{1}{{\left(\frac{\alpha }{2}-(k-1)\Delta x\right)}^{2}{\left(x+x_{k}\right)}^{2}+\alpha^{2}x_{k}^{2}}$$

This sum has many $k_{s}$, and therefore expanding it via iterating becomes difficult and cumbersome. As $\left\{x_{k}\}\leq x\leq \{x_{k}\right\}$ ∀k∈N and the interval $\left[x-\frac{\alpha }{2},x+\frac{\alpha }{2}\right]$ is infinitesimally small, which is sufficient to scalarize $\left(x+x_{k}\right)$ by 2*x* which further yields,

(A.9) $$\sum_{k=1}^{n}\frac{1}{{\left(\frac{\alpha }{2}-(k-1)\Delta x\right)}^{2}{\left(x+x_{k}\right)}^{2}+\alpha^{2}x_{k}^{2}}=\sum_{k=1}^{n}\frac{1}{2\alpha^{2}x^{2}+4x^{2}\Delta x(k-1)\{\Delta x(k-1)-\alpha \}}$$

Let $A=2\alpha^{2}x^{2}$ and $B^{⁎}=4x^{2}\Delta x(k-1)\{\Delta x(k-1)-\alpha \}$. Now iterating *k* has only effect on $B^{⁎}$. That means when,

k=1; $B^{⁎}=0$

k=2; $B^{⁎}=4x^{2}\Delta x(\Delta x-\alpha )=B(say)$

k=3; $B^{⁎}=8x^{2}\Delta x(2\Delta x-\alpha )=2B(1+\frac{\Delta x}{\Delta x-\alpha })$

k=4; $B^{⁎}=12x^{2}\Delta x(3\Delta x-\alpha )=3B(1+\frac{2\Delta x}{\Delta x-\alpha })$

k=5; $B^{⁎}=16x^{2}\Delta x(4\Delta x-\alpha )=4B(1+\frac{3\Delta x}{\Delta x-\alpha })$

and so on....

Generalizing this pattern, the sum is expressed in terms of *A* and *B* as,

(A.10) $$\sum_{k=1}^{n}\frac{1}{2\alpha^{2}x^{2}+4x^{2}\Delta x(k-1)\{\Delta x(k-1)-\alpha \}}=\sum_{k=1}^{n}\frac{1}{A+(k-1)B[1+\frac{(k-2)\Delta x}{\Delta x-\alpha }]}$$

Using similar argument, the denominator of the second term becomes,

(A.11) $$\sum_{k=1}^{n}\frac{1}{{\left(x^{2}-x_{0}^{2}\right)}^{2}+\alpha^{2}x_{0}^{2}}=\frac{n}{A}$$

Thus, the second term of the inequality reduces to,

(A.12) $$\frac{1}{n}\sum_{k=1}^{n}\frac{1}{1+(k-1)\frac{B}{A}[1+\frac{(k-2)\Delta x}{\Delta x-\alpha }]}$$

It can be seen that the second term is now independent of *x*, and under the limit n→∞ or Δx→0, and using the relation $\Delta x=\frac{\alpha }{n}$, one can easily reduce the second term as,

(A.13) $$\frac{1}{n}\sum_{k=1}^{n}\frac{1}{1+(k-1)\frac{B}{A}[1+\frac{(k-2)\Delta x}{\Delta x-\alpha }]}=\sum_{k=1}^{n}\frac{n}{{\left(n-k\right)}^{2}+k^{2}}$$

The term

$$\sum_{k=1}^{n}\frac{n}{{\left(n-k\right)}^{2}+k^{2}}$$

can be expanded as a geometric series,

(A.14) $$\lim_{n\to \infty }\frac{1}{n}\sum_{k=1}^{n}\{1+{\left[\frac{2(n-k)k}{n^{2}}\right]}^{1}+{\left[\frac{2(n-k)k}{n^{2}}\right]}^{2}+{\left[\frac{2(n-k)k}{n^{2}}\right]}^{3}+.....\}$$

After evaluating the limit, the series terms are as follows,

The first term is $\frac{2^{0}}{1}\left(\begin{array}{c} 0\\ 0 \end{array}\right)$

The second term is $\frac{2^{0}}{1}\left(\begin{array}{c} 0\\ 0 \end{array}\right)+\frac{2^{1}}{2}\left(\begin{array}{c} 1\\ 0 \end{array}\right)-\frac{2^{1}}{3}\left(\begin{array}{c} 1\\ 1 \end{array}\right)$

The third term is $\frac{2^{0}}{1}\left(\begin{array}{c} 0\\ 0 \end{array}\right)+\frac{2^{1}}{2}\left(\begin{array}{c} 1\\ 0 \end{array}\right)-\frac{2^{1}}{3}\left(\begin{array}{c} 1\\ 1 \end{array}\right)+\frac{2^{2}}{3}\left(\begin{array}{c} 2\\ 0 \end{array}\right)-\frac{2^{2}}{4}\left(\begin{array}{c} 2\\ 1 \end{array}\right)+\frac{2^{2}}{5}\left(\begin{array}{c} 2\\ 2 \end{array}\right)$

The fourth term is $\frac{2^{0}}{1}\left(\begin{array}{c} 0\\ 0 \end{array}\right)+\frac{2^{1}}{2}\left(\begin{array}{c} 1\\ 0 \end{array}\right)-\frac{2^{1}}{3}\left(\begin{array}{c} 1\\ 1 \end{array}\right)+\frac{2^{2}}{3}\left(\begin{array}{c} 2\\ 0 \end{array}\right)-\frac{2^{2}}{4}\left(\begin{array}{c} 2\\ 1 \end{array}\right)+\frac{2^{2}}{5}\left(\begin{array}{c} 2\\ 2 \end{array}\right)+\frac{2^{3}}{4}\left(\begin{array}{c} 3\\ 0 \end{array}\right)-\frac{2^{3}}{5}\left(\begin{array}{c} 3\\ 1 \end{array}\right)+\frac{2^{3}}{6}\left(\begin{array}{c} 3\\ 2 \end{array}\right)-\frac{2^{3}}{7}\left(\begin{array}{c} 3\\ 3 \end{array}\right)$

The fifth term is $\frac{2^{0}}{1}\left(\begin{array}{c} 0\\ 0 \end{array}\right)+\frac{2^{1}}{2}\left(\begin{array}{c} 1\\ 0 \end{array}\right)-\frac{2^{1}}{3}\left(\begin{array}{c} 1\\ 1 \end{array}\right)+\frac{2^{2}}{3}\left(\begin{array}{c} 2\\ 0 \end{array}\right)-\frac{2^{2}}{4}\left(\begin{array}{c} 2\\ 1 \end{array}\right)+\frac{2^{2}}{5}\left(\begin{array}{c} 2\\ 2 \end{array}\right)+\frac{2^{3}}{4}\left(\begin{array}{c} 3\\ 0 \end{array}\right)-\frac{2^{3}}{5}\left(\begin{array}{c} 3\\ 1 \end{array}\right)+\frac{2^{3}}{6}\left(\begin{array}{c} 3\\ 2 \end{array}\right)-\frac{2^{3}}{7}\left(\begin{array}{c} 3\\ 3 \end{array}\right)+\frac{2^{4}}{5}\left(\begin{array}{c} 4\\ 0 \end{array}\right)-\frac{2^{4}}{6}\left(\begin{array}{c} 4\\ 1 \end{array}\right)+\frac{2^{4}}{7}\left(\begin{array}{c} 4\\ 2 \end{array}\right)-\frac{2^{4}}{8}\left(\begin{array}{c} 4\\ 3 \end{array}\right)+\frac{2^{4}}{9}\left(\begin{array}{c} 4\\ 4 \end{array}\right)$

and following this pattern it is easy to calculate that the sum of higher term of series converges to $\frac{\pi }{2}$. These series terms can be written into more compact form as,

(A.15) $$\sum_{k=1}^{\infty }\{\sum_{r=0}^{k-1}\frac{2^{k-1}{\left(-1\right)}^{r}}{k+r}\left(\begin{array}{c} k-1\\ r \end{array}\right)\}=\frac{\pi }{2}$$

Hence, it is shown,

(A.16) $$\lim_{n\to \infty }\sum_{k=1}^{n}\frac{1}{{\left(x^{2}-x_{k}^{2}\right)}^{2}+\alpha^{2}x_{k}^{2}}=\frac{\pi }{2}\lim_{n\to \infty }\sum_{k=1}^{n}\frac{1}{{\left(x^{2}-x_{o}^{2}\right)}^{2}+\alpha^{2}x_{o}^{2}}$$

## Appendix B Numerical proof

Here, it will be shown numerically. Assume that x=1000 and α=0.0001. The interval would therefore be $\left[1000-\frac{0.0001}{2},1000+\frac{0.0001}{2}\right]$. Insert n = 200000 points to discretize this interval. Then any point in the interval, say, $x_{k}$ can be found as $x_{k}=x_{0}+(k-1)\frac{\alpha }{n}$. With this much information, it is possible to compute the sum of the following series numerically:

$Series1=\sum_{k=1}^{n}\frac{1}{{\left(x^{2}-x_{k}^{2}\right)}^{2}+\alpha^{2}x_{k}^{2}}$

$Series2=\sum_{k=1}^{n}\frac{1}{{\left(x^{2}-x_{0}^{2}\right)}^{2}+\alpha^{2}x_{0}^{2}}$

$Series3=\sum_{k=1}^{n}\frac{1}{\alpha^{2}x^{2}}$

$Series4=\sum_{k=1}^{n}\frac{1}{\alpha^{2}x_{0}^{2}}$

The calculated series sum are shown in Fig. B.2 for k=1 to 200000, along with their series ratio as illustrated by inequalities (A.2), (A.7). Similarly, for the same values of *α* and *n*, the following series sum are computed, as shown in Fig. B.3.

$Series5=\sum_{k=1}^{n}\frac{n}{{\left(n-k\right)}^{2}+k^{2}}$

$Series6=\sum_{k=1}^{n}\frac{n}{n^{2}+k^{2}}$

$Series7=\sum_{k=1}^{n}\frac{2n}{n^{2}+k^{2}}$

One can notice from Appendix A that Series 5 is independent of *x*. Furthermore, it has shown that for larger *n*, Series 5 converges to $\frac{\pi }{2}$. As an corollary of Series 5, it is important to notice that Series 6 represents the famous Gregory - Leibniz - Madhava series and therefore converges to $\frac{\pi }{4}$. Finally, the series $\sum_{k=1}^{\infty }\{\sum_{r=0}^{k-1}\frac{2^{k-1}{\left(-1\right)}^{r}}{k+r}\left(\begin{array}{c} k-1\\ r \end{array}\right)\}$ and Gregory - Leibniz - Madhava series $\sum_{k=1}^{\infty }\frac{{\left(-1\right)}^{k+1}}{2k-1}$ are shown in Fig. B.4. One can easily notice that the convergence rate of the former series is higher than the latter. The calculated value of pi in MATLAB using long display format, $\sum_{k=1}^{500}\{\sum_{r=0}^{k-1}\frac{2^{k-1}{\left(-1\right)}^{r}}{k+r}\left(\begin{array}{c} k-1\\ r \end{array}\right)\}$ is 3.1415926535897932384626433832795.

## Appendix C

Table C.2 contains the information required to create Fig. 1. The first and second columns reflect data obtained by Grimes and Cowley, while the third column is computed using Equation (18).
